# Acupuncture for chronic insomnia with mild cognitive impairment: protocol for a randomized controlled trial

**DOI:** 10.3389/fneur.2026.1790480

**Published:** 2026-06-19

**Authors:** Xueqian Jia, Gongyue Zhou, Pei Shen, Sangyi Lin, Haijiang Zhou, Fu Xu, Shuo Jiang, Cong Wang

**Affiliations:** 1 The First School of Clinical Medicine, Zhejiang Chinese Medical University, Hangzhou, China; 2 Department of Acupuncture and Moxibustion, The First Affiliated Hospital of Zhejiang Chinese Medical University (Zhejiang Provincial Hospital of Chinese Medicine), Hangzhou, China; 3 Department of Preventive Treatment, The First Affiliated Hospital of Zhejiang Chinese Medical University (Zhejiang Provincial Hospital of Chinese Medicine), Hangzhou, China

**Keywords:** a randomized controlled trial, acupuncture, chronic insomnia, mild cognitive, protocol

## Abstract

**Introduction:**

Chronic insomnia frequently coexists with mild cognitive impairment (MCI) and may aggravate cognitive and daytime dysfunction. Pharmacological treatments can cause adverse effects, and cognitive behavioral therapy for insomnia (CBT-I) may be limited by resource and accessibility barriers. Acupuncture is a commonly used non-pharmacological intervention, but sham-controlled evidence in patients with chronic insomnia and comorbid MCI remains limited. This protocol describes, to our knowledge, one of the few sham-controlled randomized trials specifically targeting this comorbid population.

**Methods and analysis:**

This is a single-center, participant- and assessor-blinded, sham-controlled, parallel-group randomized clinical trial. Eighty-two participants with chronic insomnia and MCI will be randomized in a 1:1 ratio to an acupuncture group or a sham-acupuncture group. Both groups will receive 10 sessions over approximately 4 weeks, followed by 1-week and 1-month follow-up assessments; total participation will last approximately 8–9 weeks. The single primary endpoint is the between-group difference in change in Pittsburgh Sleep Quality Index (PSQI) total score from baseline to post-treatment (week 4). Key secondary outcomes are the Insomnia Severity Index (ISI), Montreal Cognitive Assessment-Basic (MoCA-B), and Mini-Mental State Examination (MMSE). Other secondary outcomes include polysomnography (PSG), activities of daily living (ADL), executive-function tests (DST, SCWT, TMT), mood symptoms (BAI, BDI), daytime functioning (FSS, ESS), feasibility/acceptability indices, and safety. Resting-state electroencephalography (EEG) outcomes are exploratory. The primary analysis will use the full analysis set and a repeated-measures linear mixed-effects model.

**Clinical trial registration:**

International Traditional Medicine Clinical Trial Registry (http://itmctr.ccebtcm.org.cn/), Identifier ITMCTR2025002221.

## 1 Introduction

Chronic insomnia is a prevalent clinical disorder characterized by persistent difficulties in initiating or maintaining sleep, accompanied by impaired daytime functioning. A substantial portion of the global population is affected, with an estimated prevalence of approximately 16.2%; the condition is more prevalent in women and older adults (1). A recent large-scale epidemiological survey in China reported an even higher insomnia prevalence rate of 24.8% (2), with elevated rates observed in women, highly educated individuals, and the elderly. A multi-center cross-sectional study found a clinical insomnia prevalence rate of 28.9% among individuals aged 60 and above (3). Beyond its core symptoms, insomnia is associated with an increased risk of various physical conditions, including cardiovascular disease, kidney disease, and neurological disorders, with particularly pronounced effects on psychiatric health. Substantial evidence demonstrates a robust association between insomnia and the development of anxiety and depression, conditions that exhibit high comorbidity rates and functional impairment (4). Notably, insomnia can hinder recovery in individuals with depression, while residual insomnia following depression treatment increases the risk of relapse (5). In addition to causing significant physical harm, insomnia also impairs social functions, thereby increasing socio-economic burdens.

Cognitive impairment encompasses a range of abnormal symptoms arising from damage to higher brain functions caused by diverse etiologies. Subjective cognitive decline is most prevalent among the elderly and is frequently age-related. Cognitive impairment affects patients’ daily activities and social functions, and it is frequently associated with common complications including affective disorders (e.g., anxiety and depression), behavioral changes (e.g., apathy and agitation), and a decline in the instrumental activities of daily living. Surveys indicate that the global dementia population is expected to reach 150 million by 2050 (6). Increasing evidence indicates that the occurrence and progression of chronic insomnia are associated with an elevated risk of cognitive impairment (7, 8). Mild cognitive impairment (MCI), as a prodromal stage of dementia, represents an intermediate state between normal aging and dementia (9). Emerging evidence suggests a bidirectional relationship between chronic insomnia and MCI, with each condition potentially exacerbating the other. One study found that sleep disorders increased the risk of developing MCI by 2.2 times over 4 years (10), while another study reported that individuals with a history of sleep disorders had an odds ratio (OR) of 1.956 for developing MCI, particularly for a history of chronic insomnia, the OR reached 1.910 (11, 12). MCI-phase interventions are therefore critical for preventing or delaying the onset of more severe cognitive impairment. This is especially relevant for patients with chronic insomnia comorbid with MCI, as treatment must simultaneously address both insomnia symptoms and cognitive dysfunction, increasing therapeutic complexity and requiring heightened clinical attention.

The current clinical management of chronic insomnia primarily encompasses pharmacological and non-pharmacological approaches. Among non-pharmacological therapies, cognitive behavioral therapy for insomnia (CBT-I) is internationally recognized as the first-line treatment. It primarily works by modifying patients’ maladaptive thoughts, beliefs, and behaviors concerning sleep, thus alleviating negative emotions and behaviors (13). Research has confirmed that CBT-I significantly improves both insomnia severity and co-occurring negative emotional states (5). However, CBT-I requires extended treatment cycles, demands highly trained therapists, and involves relatively high costs, limiting its widespread application in domestic clinical settings. Regarding pharmacotherapy, benzodiazepines, non-benzodiazepine hypnotics, and melatonin receptor agonists are commonly utilized clinically. Other medications such as antihistamines, antidepressants, antiepileptics, and antipsychotics are also sometimes employed off-label for insomnia. While pharmacotherapy offers short-term benefits, long-term use carries risks of dependence, tolerance, and addiction. With advancing age, age-related changes in pharmacokinetics and pharmacodynamics increase the potential for inappropriate use of medication and multiple adverse effects (14, 15).

Management of patients with MCI primarily relies on non-pharmacological interventions, with medications often serving merely as adjunctive therapies. In recent years, non-pharmacological approaches to MCI have gained prominence, including cognitive interventions, dietary and exercise modifications, and traditional Chinese medicine. Cognitive training is currently the preferred non-pharmacological treatment (16), which can be classified into traditional cognitive training and computerized cognitive training (CCT). Studies have indicated that CCT improves overall cognition, memory, working memory, and attention in MCI patients, while also enhancing psychosocial functions (17). However, it exhibits limited benefits for other cognitive domains including executive function and processing speed. Some progress has been made in pharmacological interventions for MCI, particularly with monoclonal antibodies targeting *β*-amyloid, which demonstrates efficacy in slowing clinical progression in specific MCI (18). While statistically significant, these therapies offer only modest cognitive gains (e.g., 0.5–1.5 points), which are often regarded clinically insignificant. This marginal benefit, weighed against the risks of amyloid-related imaging abnormalities with edema (ARIA-E) or hemosiderin deposition (ARIA-H), the burden of regular MRI monitoring, and the high costs, has prevented widespread application (19).

Acupuncture, a traditional non-pharmacological therapy, is increasingly applied in clinical practice considering its characteristics of being simple, effective, and rapid-acting. Previous clinical studies have suggested that acupuncture may improve subjective sleep quality and related symptoms in patients with insomnia, although the certainty and generalizability of the evidence vary across study designs (20). Regarding comorbid insomnia and depression, studies have confirmed that acupuncture reduces hyperactivity of the hypothalamic–pituitary–adrenal axis, restoring neuroendocrine rhythms, and subsequently improving both mood and sleep (21). Furthermore, a randomized clinical trial treating insomnia in depressed patients demonstrated that the acupuncture group showed significantly greater improvement in sleep quality by week 8 compared to the medication group, with effects sustained at week 32, indicating the durable efficacy of acupuncture for insomnia (22). Acupuncture also shows therapeutic potential for MCI. A preliminary randomized, patient and examiner-blinded, sham-controlled study found that acupuncture improves cognitive function in MCI patients and shows potential for enhancing memory (23). Different timing and methods of acupuncture administration have been shown to yield different degrees of improvement in MCI patients (24). However, to date, only one study has specifically investigated the efficacy of acupuncture for the comorbidity of insomnia and cognitive impairment (25). That previous study had several critical limitations, such as the absence of a sham-acupuncture control, a small sample size, lack of randomization, and insufficient objective measures. Therefore, to address these methodological limitations and provide high-quality evidence, the present RCT was designed.

The present study was designed to address this evidence gap using a sham-controlled randomized clinical trial in patients with chronic insomnia comorbid with MCI. The principal hypothesis is that acupuncture will produce a greater reduction in PSQI total score from baseline to post-treatment (week 4) than sham acupuncture. Secondary objectives are to evaluate insomnia severity, global cognition, objective sleep architecture, mood symptoms, executive function, daytime fatigue/sleepiness, feasibility/acceptability, and safety. Resting-state EEG measures are included as exploratory mechanistic outcomes rather than confirmatory efficacy outcomes.

## 2 Methods

### 2.1 Study design

This is a single-center, participant- and assessor-blinded, sham-controlled, parallel-group randomized clinical trial conducted at the Zhejiang Provincial Hospital of Traditional Chinese Medicine. The study is scheduled to run from January 2025 to December 2027 under the administration of the Zhejiang Provincial Administration of Traditional Chinese Medicine. Eighty-two participants with chronic insomnia comorbid with MCI will be allocated in a 1:1 ratio to acupuncture or sham acupuncture, with 41 participants in each group.

The principal clinical hypothesis is that acupuncture will produce a greater reduction in PSQI total score from baseline to post-treatment (week 4, after the tenth treatment session) than sham acupuncture. The primary objective is therefore to evaluate the efficacy of acupuncture on subjective sleep quality. Key secondary objectives are to evaluate insomnia severity and global cognitive function. Other secondary objectives include objective sleep architecture, daily functioning, executive function, mood symptoms, daytime function, feasibility/acceptability, and safety. EEG outcomes are exploratory and will be used to generate mechanistic hypotheses.

**Figure 1:**
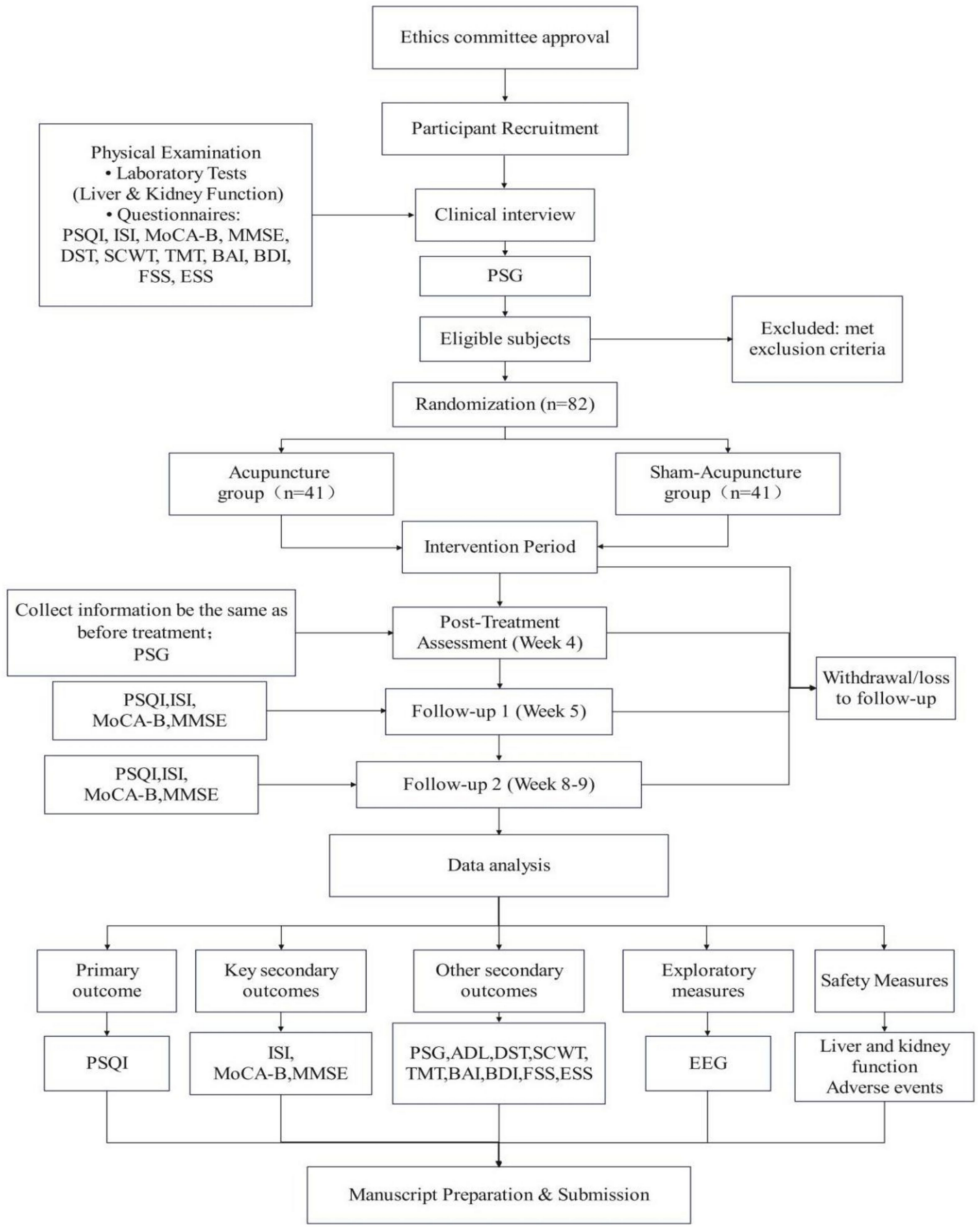
Flow diagram. ADL, activities of daily living; BAI, Beck anxiety inventory; BDI, Beck depression inventory; EEG, resting-state electroencephalography; ESS, Epworth Sleepiness Scale; FSS, Fatigue Severity Scale; ISI, Insomnia Severity Index; MMSE, mini-mental state examination; MOCA-B, montreal cognitive assessment-basic; PSG, polysomnography; PSQI, Pittsburgh Sleep Quality Index; SCWT, stroop color word test; TMT, trail making test.

Each participant will progress through three phases: screening and baseline assessment, a 4-week treatment period, and follow-up assessments at 1 week and 1 month after treatment. The total participation period for each participant will be approximately 8–9 weeks. The participant timeline is presented in Table 1, and the trial flow is shown in Figure 1.

**Table 1:** Participant timeline and assessment schedule.

Week	0	1	2	3	4	5	8–9
Baseline period		Intervention treatment period				Follow-up 1 week	Follow-up 1 month
Patients							
Preliminary screening	**×**						
Outpatient interview assessment	**×**						
Signing of informed consent	**×**						
Scale assessment	**×**						
Physical examination	**×**				**×**		
Laboratory tests	**×**				**×**		
PSG	**×**				**×**		
EEG	**×**				**×**		
Group							
Acupuncture group	10 treatments						
Sham-acupuncture group	10 treatments						
Outcome measurement							
PSQI	**×**				**×**	**×**	**×**
ISI	**×**				**×**	**×**	**×**
MoCA-B	**×**				**×**	**×**	**×**
MMSE	**×**				**×**	**×**	**×**
ADL	**×**				**×**		
DST	**×**				**×**		
SCWT	**×**				**×**		
TMT	**×**				**×**		
BAI	**×**				**×**		
BDI	**×**				**×**		
FSS	**×**				**×**		
ESS	**×**				**×**		
CEQ		**×**			**×**		
Sleep diary	**×**	**×**	**×**	**×**	**×**	**×**	**×**
Adverse events	**×**	**×**	**×**	**×**	**×**	**×**	**×**
Withdrawal/loss to follow-up and reasons		**×**	**×**	**×**	**×**	**×**	**×**

ADL, activities of daily living; BAI, Beck anxiety inventory; BDI, Beck depression inventory; CEQ, credibility expectancy questionnaire; DST, digit span test; EEG, resting-state electroencephalography; ESS, Epworth Sleepiness Scale; FSS, Fatigue Severity Scale; ISI, Insomnia Severity Index; MMSE, mini-mental state examination; MOCA-B, montreal cognitive assessment-basic; PSG, polysomnography; PSQI, Pittsburgh Sleep Quality Index; SCWT, stroop color word test; TMT, trail making test.

Data for the primary endpoint and key secondary outcomes (PSQI, ISI, MoCA-B, and MMSE) will be collected at baseline, immediately after treatment completion (week 4), at the 1-week follow-up (week 5), and at the 1-month follow-up (week 8–9). PSG, EEG, ADL, executive-function tests, BAI, BDI, FSS, and ESS will be assessed at baseline and immediately after treatment. Participants will maintain a sleep diary throughout the treatment and follow-up periods under physician guidance.

### 2.2 Participants

This study will enroll 82 adult patients diagnosed with chronic insomnia and MCI. Recruitment will be conducted at Zhejiang Provincial Hospital of Traditional Chinese Medicine, supplemented by social media and public advertisements. Interested individuals will contact the research coordinator by telephone and will then attend an outpatient screening visit. All clinical procedures will be performed within the Acupuncture Department of the hospital.

### 2.3 Inclusion criteria

The diagnosis of insomnia is based on criteria for chronic insomnia disorder as defined by the International Classification of Sleep Disorders, Third Edition (ICSD-3) (26). The diagnosis of MCI adhered to the National Institute on Aging and Alzheimer’s Association (NIA-AA) criteria (27) and the Chinese Guidelines for the Diagnosis and Treatment of Dementia and Cognitive Disorders (V): Diagnosis and Treatment of MCI (28). Eligible participants should meet all of the following criteria:

1. Meets the ICSD-3 diagnostic criteria for chronic insomnia disorder and has clinically significant insomnia symptoms, defined as ISI ≥ 15 and PSQI total score > 5.
2. Meets the diagnostic criteria for MCI according to the NIA-AA framework and Chinese guidelines: subjective cognitive complaint; objective cognitive impairment based on education-adjusted MoCA-B cut-offs (≤ 13 for no formal education, ≤ 19 for 1–6 years of education, and ≤ 24 for ≥ 7 years of education); essentially preserved activities of daily living (ADL ≤ 25); Clinical Dementia Rating (CDR) score ≤ 0.5; and does not meet the diagnostic criteria for dementia (29). The MMSE will be used as a supportive global cognitive assessment and to help exclude dementia according to education-adjusted clinical judgment rather than as a stand-alone unadjusted inclusion threshold.
3. Aged 18 years or older.
4. Right-handed, with sufficient hearing, vision, sensory function, and comprehension to complete the study procedures.
5. No use of sedative-hypnotic medication, sleep aid, CBT-I, acupuncture for insomnia or cognitive impairment, or other sleep-targeted treatment for at least 4 weeks before enrolment.
6. Provision of written informed consent before any study-specific procedure.

### 2.4 Exclusion criteria

1. Significant impairment of cardiac, hepatic, renal, respiratory, or hematologic function, or any unstable systemic disease judged by the investigator to render participation unsafe.
2. Other sleep disorders confirmed by clinical interview or PSG, including sleep apnea with apnea-hypopnea index ≥ 10 events/h or periodic limb movements during sleep > 15 events/h.
3. Acute or unstable neurological disease, including acute stroke, severe traumatic brain injury, or other neurological onset within the previous 6 months.
4. Severe neurological deficits, major physical functional impairment, or structural brain lesions on imaging that are likely to explain cognitive impairment, including hydrocephalus, tumor, or other degenerative patterns inconsistent with the intended MCI population.
5. Current major psychiatric disorder, psychosis, substance-use disorder, or severe depressive/anxiety episode requiring urgent treatment; mild mood symptoms commonly associated with chronic insomnia will be recorded and analyzed rather than automatically excluded.
6. Current or recent use of medications or interventions likely to confound sleep or cognitive outcomes, including sedative-hypnotics, antipsychotics, cholinesterase inhibitors initiated or changed recently, CBT-I, acupuncture for insomnia/MCI, or participation in other interventional clinical research within the past 3 months.
7. Pregnancy or lactation.
8. Infectious diseases including active or uncontrolled infectious disease posing a procedural or infection-control risk.
9. Contraindications to acupuncture, including unhealed wounds at or near acupoints, severe skin disease, bleeding tendency, metal allergy, or other conditions judged unsuitable by the investigator.
10. Inability to complete follow-up assessments or to comply with study procedures.

### 2.5 Interventions

The acupuncturists performing treatments will hold Master’s degrees in Acupuncture and Tuina Therapy from Zhejiang Chinese Medical University, physician qualification certificates, and more than three years of clinical acupuncture experience. Before recruitment, all acupuncturists, assessors, and research coordinators will complete standardized training in the intervention protocol, sham procedure, participant communication, adverse-event recording, and blinding protection. The protocol is reported in accordance with the Standard Protocol Items: Recommendations for Interventional Trials SPIRIT (2025) statement, and the acupuncture intervention is described according to the Revised Standards for Reporting Interventions in Clinical Trials of Acupuncture (STRICTA) 2010 reporting guidelines (30). A completed SPIRIT 2025 checklist is provided as Supplementary material.

Participants in the acupuncture group will be placed in the supine position. After routine skin disinfection with 75% medical alcohol, sterile disposable filiform needles (0.25 × 40 mm, Asia-Med, Suhl, Germany) will be used. The acupoint prescription is based on previous systematic reviews, clinical studies, and clinical experience (31–33), and includes Baihui (GV20), Sishencong (EX-HN1), Fengchi (GB20), Anmian (Extra), Shenting (GV24), Yintang (GV29), Neiguan (PC6), Shenmen (HT7), Sanyinjiao (SP6), Xuanzhong (GB39), Shenmai (BL62), and Zhaohai (KI6). Bilateral acupoints will be used where applicable. Needles will be inserted to a depth of approximately 10–20 mm according to body habitus and acupoint location. Manual lifting, thrusting, and twirling will be applied to elicit de qi, and needles will be retained for 30 min.

Participants in the sham-acupuncture group will receive a non-penetrating placebo needle (Streitberger device) at acupoint locations identical to those used in the acupuncture group. The Streitberger placebo needle has been widely used in acupuncture trials (34). For scalp or hair-bearing regions, surgical tape or hairpins will be used to maintain the appearance and stability of the needle device. No skin penetration or manipulation intended to elicit de qi will be performed in the sham group. Both groups will receive the same number of sessions, treatment duration, acupoint locations, room setting, and patient-practitioner interaction time to minimize expectation and performance bias.

Both groups will receive a total of 10 treatment sessions delivered over approximately 3 to 4 weeks, at a frequency of 2 to 3 sessions per week on non-consecutive days. To maintain participant blinding, all participants will be told using a standardized script: “This study compares two forms of acupuncture used in clinical research. Because both procedures are credible acupuncture-related interventions, participants will not be told which form is expected to be superior.” Participants will be instructed not to discuss treatment sensations or perceived group assignment with outcome assessors.

### 2.6 Outcome measures

#### 2.6.1 Primary outcome

The PSQI is a 19-item self-rated questionnaire grouped into seven components: subjective sleep quality, sleep latency, sleep duration, habitual sleep efficiency, sleep disturbances, use of sleep medication, and daytime dysfunction. The global score ranges from 0 to 21, with higher scores indicating poorer sleep quality (35). A score >5 is widely used to identify clinically significant poor sleep, and the Chinese version has demonstrated acceptable reliability and validity (36). The single primary endpoint is the between-group difference in change in PSQI total score from baseline to week 4. The PSQI will be administered at baseline, week 4, week 5, and week 8–9.

#### 2.6.2 Key secondary outcomes

The ISI is a 7-item self-report instrument assessing nighttime and daytime components of insomnia over the preceding 2 weeks (37). Scores range from 0 to 28, with higher scores indicating greater insomnia severity; scores ≥ 15 indicate clinically significant insomnia. The ISI will be administered at baseline, week 4, week 5, and week 8–9.

The MoCA-B assesses global cognition across domains including executive function, memory, attention, language, visuoconstructional skills, conceptual thinking, calculation, and orientation (38). The following education-adjusted cut-off scores are used to define cognitive impairment: ≤ 13 for individuals with no formal education, ≤ 19 for those with 1–6 years of education, and ≤ 24 for those with ≥ 7 years of education. The MoCA-B will be administered at baseline, week 4, week 5, and week 8–9.

The MMSE is a global cognitive screening instrument assessing orientation, calculation, memory, language, repetition, and visuoconstructional ability (39). Education-adjusted interpretation will be used, and MMSE results will support clinical characterization and dementia exclusion rather than serving as an independent primary endpoint (40). The MMSE will be administered at baseline, week 4, week 5, and week 8–9.

#### 2.6.3 Other secondary outcomes

Secondary outcomes encompass objective insomnia assessment (PSG), ADL, cognitive functions and executive ability assessment (DST, SCWT, and TMT), emotional state assessment (BAI, and BDI), daytime functioning assessment (FSS, and ESS), and exploratory measures (EEG) (Table 1).

##### 2.6.3.1 Objective sleep assessment

Objective sleep will be assessed using PSG at baseline and week 4. The PSG system (NIHON KOHDEN) will record EEG, mandibular electromyography, bilateral electrooculography, oral/nasal airflow, and bilateral tibialis anterior electromyography. Two consecutive nights of PSG will be collected before and after treatment to reduce the first-night effect (41). Each monitoring session will begin at the participant’s habitual bedtime and continue for a total recording time of approximately 8 h. Full-night sleep EEG will be recorded in 30-s epochs, with sleep stages and respiratory events manually scored according to the American Academy of Sleep Medicine Manual for the Scoring of Sleep and Associated Events (Version 2.6) (42). Objective sleep parameters include: Total Sleep Duration (TST); REM Sleep Latency; Sleep Onset Latency (SOL: lights out to first epoch of any sleep); Wake After Sleep Onset (total recording time minus SOL minus TST); Sleep efficiency (TST / total recording time × 100%); Arousal index (Arl; arousal count × 60 / TST); Frequency of wakening; Percent of TST in each stage (stage duration / TST × 100%).

##### 2.6.3.2 Activities of daily living

Activities of daily living will be assessed using the revised Chinese ADL scale (43). The scale comprises two domains: basic ADL and instrumental ADL. The former includes eating, dressing/undressing, personal hygiene, getting in/out of bed and sitting/standing, walking indoors, using the toilet, urinary/fecal control, and bathing. The latter encompasses riding public transportation, outdoor activities, cooking for oneself, taking medication on time, light housework, heavy housework, washing one’s own clothes, cutting toenails, shopping, using the telephone, managing personal finances, and staying home alone for a day. Each item offers four response options: “Can do completely independently,” “Can do with some difficulty,” “Needs assistance,” and “Cannot do at all,” corresponding to scores of 1, 2, 3, and 4, respectively. The total score ranges from 20 to 80 points, with 20–25 points indicating normal daily living abilities and 26 points or higher demonstrating impaired daily living ability (44).

##### 2.6.3.3 Cognitive executive functions

Working memory will be assessed using the Digit Span Test (DST) (45). Participants will be asked to repeat sequences of digits in forward and in reverse order, with the longest correctly repeated span recorded separately for each condition. All neuropsychological tests will be administered by the same trained graduate student using standardized procedures.

Inhibitory control will be assessed using the Stroop Color Word Test (SCWT) (46). The test comprises three sets of cards, each containing 50 stimuli: (a) Chinese characters denoting colours printed in black ink; (b) coloured circles in red, yellow, blue, or green; and (c) Chinese colour words printed in incongruent ink colours. Participants will be instructed to name the character, the colour of the circle, and the ink colour of the printed word, respectively. The time required to complete each card and the number of correct responses will be recorded.

Cognitive flexibility and processing speed will be assessed using the Trail Making Test (TMT) (47). Participants will connect the dots on TMT Card A in numerical order from 1 to 25, and on TMT Card B in alternating order between numbers and Chinese characters. Time to completion will be recorded for each card.

##### 2.6.3.4 Emotional state

Anxiety symptoms will be assessed using the Beck Anxiety Inventory (BAI), a 21-item self-report scale on which higher total scores indicate greater anxiety severity (48). It assesses anxiety symptoms over the preceding 7 days. The standard interpretation defines scores of 0–7 as minimal, 8–15 as mild, 16–25 as moderate, and 26–63 as severe anxiety.

Depressive symptoms will be assessed using the Beck Depression Inventory (BDI), originally developed by Beck based on the diagnostic criteria listed in the Diagnostic and Statistical Manual of Mental Disorders (4th Ed.) (DSM-IV) (49). The BDI comprises 21 self-report items rated for the preceding 7 days; higher total scores indicate greater depression severity (0–13 minimal, 14–19 mild, 20–28 moderate, and 29–63 severe).

##### 2.6.3.5 Daytime functioning

Daytime fatigue will be assessed using the Fatigue Severity Scale (FSS), a 9-item self-report instrument originally developed by Krupp et al. (50). Each item is rated on a 7-point Likert scale, and the total score is calculated as the mean of all nine items, with higher scores indicating greater fatigue. A mean FSS score ≥ 4 is commonly used to identify clinically significant fatigue.

Daytime sleepiness will be assessed using the Epworth Sleepiness Scale (ESS), an 8-item self-report scale developed by Johns (51). Each item rates the likelihood of dozing in a typical daily situation (reading, watching television, sitting inactive in a public place, riding as a passenger for an hour, lying down to rest in the afternoon, sitting and talking, sitting quietly after lunch, and stopping briefly in traffic) on a scale of 0 (no chance) to 3 (high chance). The total score ranges from 0 to 24, with higher scores indicating greater daytime sleepiness.

Feasibility and acceptability outcomes will include the recruitment rate, treatment adherence (number of completed treatment sessions), retention at each follow-up visit, completion of outcome assessments, reasons for withdrawal, use of prohibited or rescue sleep-related treatments, and participant blinding responses. To address potential cultural expectancy effects, treatment credibility and outcome expectancy will be assessed after the first treatment session and after the final treatment session using the Credibility Expectancy Questionnaire (CEQ) (52); between-group differences in CEQ scores will be reported descriptively to evaluate the comparability of expectations between the acupuncture and sham-acupuncture groups. Adverse events and serious adverse events will be analyzed as safety outcomes.

#### 2.6.4 Exploratory measures

Resting-state EEG will be recorded at baseline and at week 4 using the international 10–20 electrode system. Cz will serve as the reference electrode, with a sampling rate of 1,000 Hz and an online high-pass filter of 0.5 Hz. Five complementary analytic approaches will be used to extract resting-state EEG features: power spectral density, frontal alpha asymmetry, non-linear neurodynamics, functional connectivity, and EEG microstate analysis. As exploratory mechanistic hypotheses, we will test whether acupuncture compared with sham is associated with (i) reductions in relative beta power and increases in relative alpha/theta power over fronto-central regions at rest, (ii) reduced frontal alpha asymmetry, and (iii) altered contributions of microstates C and D, which have been linked to attentional and arousal networks. All EEG analyses will be exploratory, will be reported with effect sizes and 95% confidence intervals, and will be corrected for multiple testing using the false-discovery-rate procedure.

#### 2.6.5 Measurement-instrument rationale

Patient-reported outcomes were selected with reference to the COSMIN framework, with attention to content validity, construct validity, reliability, responsiveness, and evidence for use in the intended population (53). COSMIN is applied here as a framework for evaluating and selecting measurement instruments rather than as a claim that the present trial independently validates these scales. The PSQI and ISI serve as the principal sleep-related patient-reported outcomes, while the BAI, BDI, FSS, and ESS serve as secondary patient-reported outcomes. The MoCA-B and MMSE are clinician-administered cognitive screening instruments and are justified separately on the basis of prior validation in Chinese and low-education populations.

#### 2.6.6 Safety assessments

Liver and kidney function will be assessed during screening and after treatment to monitor systemic safety. All adverse events (AEs) occurring from the signing of informed consent to the final follow-up visit will be prospectively recorded on a standardized AE form. The form will document the type of event, onset time, duration, severity, seriousness, relationship to the intervention, management, action taken, and outcome.

Prespecified acupuncture-related AEs include needle fainting or vasovagal symptoms, pain, needle retention, subcutaneous bleeding, hematoma, local infection, metal allergy, and any other procedure-related incident requiring medical attention. Severity will be graded using CTCAE version 5.0 when applicable: Grade 1 (mild), Grade 2 (moderate), Grade 3 (severe), Grade 4 (life-threatening), and Grade 5 (death). Causality will be categorized as definitely related, probably related, possibly related, probably unrelated, or unrelated to acupuncture.

The treating acupuncturist will report AEs immediately to the study coordinator. An independent safety monitor who is not involved in treatment or outcome assessment will review AE reports regularly. Serious adverse events (SAEs), including death, life-threatening events, hospitalization or prolonged hospitalization, persistent or significant disability, or other medically important events, will be reported to the principal investigator and the ethics committee within 24 h. Treatment discontinuation criteria include participant request, Grade 3 or higher AE judged probably or definitely related to acupuncture, SAE judged related to the intervention, serious intercurrent illness, pregnancy, or substantial protocol violation. If two or more intervention-related SAEs occur, recruitment and treatment will be suspended pending safety review. All participants who receive at least one intervention session will be included in the Safety Set.

### 2.7 Sample size

The sample size was calculated for the revised primary endpoint: the between-group difference in change in PSQI total score from baseline to post-treatment (week 4). In a previous sham-controlled acupuncture trial in chronic insomnia (54), sham acupuncture reduced the PSQI score to 14.76 ± 3.35 in insomnia patients post-treatment, with a sample standard deviation of s = 3.35. This study hypothesizes that acupuncture treatment for insomnia with MCI will yield an additional 2.70-point reduction in PSQI scores compared to sham acupuncture. Referencing the sample size estimation formula for two-sample mean comparisons in Medical Statistics (55), the calculation is as follows:


$$n=2{\left[\frac{\left(u_{\alpha }+u_{\beta }\right)}{\delta /\sigma }\right]}^{2}+\frac{1}{4}{u_{\alpha }}^{2}$$

A two-sided alpha of 0.05 and 90% power were used, with u0.05/2 = 1.96 and u0.1 = 1.282. This calculation yields n ≈ 33.32 per group, rounded up to 34. The standardised effect size δ/σ ≈ 0.81 corresponds to a large effect (Cohen’s d). Allowing for approximately 17% attrition, the final target sample size is 41 participants per group, for a total of 82 participants.

### 2.8 Randomization and blinding

An independent statistician who will not be involved in recruitment, treatment, or outcome assessment will generate the allocation sequence using SPSS version 25.0. A computer-generated 1:1 randomization sequence will be used to assign 82 participants to the acupuncture group or the sham-acupuncture group. The allocation sequence will be concealed in sequentially numbered, opaque, sealed envelopes prepared by a researcher independent of recruitment and intervention delivery. After written informed consent and completion of baseline assessment, the next envelope in sequence will be opened by the acupuncturist to determine the assigned intervention.

This trial is participant- and assessor-blinded; acupuncturists cannot be blinded because they deliver the intervention. Participant blinding will be supported by using the same acupoint locations, number of sessions, session duration, treatment environment, fixation procedures, and standardized communication in both groups. Outcome assessors and data analysts will remain blinded to group allocation and will be physically separated from the treatment area. Participants will be reminded before each assessment not to reveal treatment sensations or perceived group assignment.

The success of participant blinding will be evaluated after the final treatment session and at the final follow-up by asking participants to guess their allocation (acupuncture, sham acupuncture, or uncertain) and to rate confidence in the guess. Blinding will be summarized using the Bang blinding index and/or James blinding index. Any accidental unblinding will be documented within 24 h, including the source, timing, personnel involved, and potential effect on outcome assessment. Participants who are unblinded will remain in follow-up and in the full analysis set; if the number of unblinded participants is meaningful, sensitivity analyses excluding them will be performed. Emergency unblinding will be permitted only when knowledge of allocation is necessary for clinical management of an SAE.

### 2.9 Data collection and management

Data will be collected at baseline (within one week before the first intervention), immediately after treatment completion (week 4), at the 1-week follow-up (week 5), and at the 1-month follow-up (week 8–9). Trained outcome assessors blinded to group allocation will administer questionnaires and neuropsychological tests. Participants will complete self-report scales at the research site under standardized instructions, and a trained staff member will be available to answer procedural questions without discussing treatment allocation. Sleep diaries will be completed at home under physician guidance.

Data will be entered by trained research staff using prespecified coding rules and stored in password-protected files on a secure institutional server with restricted access. Personally identifiable information will be accessible only to essential study personnel and will be stored separately from research data. Data quality checks will be performed regularly. Participants may withdraw at any time; if a participant withdraws, the clinician will ask whether the participant is willing to complete final outcome and safety assessments, and the date and reason for withdrawal will be recorded. Protocol deviations, adherence, missing data, and use of prohibited treatments will be documented. The technical appendix, statistical code, and de-identified dataset will be available from the corresponding author upon reasonable request after completion of the study and publication of the primary results.

### 2.10 Statistics and analysis

The primary analysis will use the full analysis set, consistent with a modified intention-to-treat principle. The per-protocol set (PPS) will include participants without major protocol deviations and with adequate treatment adherence. Major protocol deviations precluding PPS inclusion include violation of enrolment criteria, use of prohibited concomitant treatments (sedative-hypnotics, CBT-I, or additional acupuncture for insomnia or MCI), missing the primary endpoint assessment by more than 7 days, and completion of fewer than 8 of the 10 planned treatment sessions. The Safety Set will include all participants who receive at least one intervention session.

The prespecified primary contrast is the between-group difference in change in PSQI total score from baseline to week 4. A repeated-measures linear mixed-effects model will be used for the primary endpoint. Fixed effects will include treatment group, visit, treatment-by-visit interaction, and the baseline PSQI value; participant will be included as a random effect. An unstructured covariance matrix will be attempted first. If convergence is unstable, alternative covariance structures, including autoregressive and compound-symmetry structures, will be compared using the Akaike information criterion.

The same mixed-effects framework will be used for longitudinal continuous key secondary outcomes (ISI, MoCA-B, and MMSE) and other continuous secondary outcomes when repeated measures are available. Outcomes assessed only at baseline and week 4 will be analyzed using analysis of covariance or linear mixed-effects models adjusted for baseline value. If model assumptions are materially violated, transformations, robust estimates, or rank-based/non-parametric analyses will be used as sensitivity analyses. Categorical outcomes will be summarized as frequencies and percentages and compared using chi-square or Fisher exact tests as appropriate. CEQ scores will be summarized descriptively and compared between groups to evaluate the comparability of treatment credibility and expectancy.

Missing outcome data in the primary analysis will be handled by likelihood-based estimation under the missing-at-random assumption. Multiple imputation by chained equations will be used as a sensitivity analysis for the primary endpoint. Participants with major protocol deviations or accidental unblinding will remain in the FAS, while PPS and sensitivity analyses will assess the robustness of the findings.

Because only one primary endpoint is prespecified, no multiplicity adjustment is required for the primary hypothesis. Secondary outcomes will be interpreted as supportive and will be adjusted within clinically related domains using the Holm procedure where appropriate. Exploratory EEG analyses will report effect sizes and 95% confidence intervals; false-discovery-rate control will be applied when multiple EEG features are tested. Two-sided tests will be used, with *p* < 0.05 considered statistically significant for the primary analysis. Prespecified exploratory subgroup analyses for the primary endpoint will include age (< 65 vs. ≥ 65 years), baseline ISI severity (moderate 15–21 vs. severe ≥ 22), and educational level (≤ 6 vs. ≥ 7 years), reported as treatment-by-subgroup interaction terms and visualised with forest plots; all subgroup analyses are explicitly exploratory and hypothesis-generating. Statistical analyses will be conducted using the SPSS MIXED procedure (version 25.0) or equivalent validated statistical software (e.g., R lme4/nlme, SAS PROC MIXED).

## 3 Discussion

Chronic insomnia and MCI frequently coexist and are clinically important because persistent sleep disruption may worsen daytime functioning, mood symptoms, and cognitive vulnerability. Insomnia is common in the general population and in older adults, including Chinese older adults (1–3). At the same time, dementia and cognitive impairment represent an increasing public-health burden (6), and accumulating evidence links insomnia or other sleep disturbances to Alzheimer’s disease-related biomarkers and to incident cognitive impairment or MCI (7, 8, 11, 12, 56, 57). Management of patients with both chronic insomnia and MCI is challenging because treatment needs to address sleep complaints, cognitive vulnerability, daytime dysfunction, and affective symptoms simultaneously. Although CBT-I is recommended as a first-line non-pharmacological treatment for chronic insomnia, access may be limited by therapist availability, treatment time, cost, and implementation barriers (13, 58, 59). Pharmacological treatment may provide short-term sleep benefits but is constrained by tolerance, dependence, inappropriate medication use, and potential cognitive and safety concerns, particularly in older adults or cognitively vulnerable patients (14, 15, 19, 60). These limitations support the need for accessible and safe non-pharmacological interventions for chronic insomnia with comorbid MCI.

Acupuncture has been investigated for insomnia and related comorbid symptoms. Previous studies and reviews suggest that acupuncture may improve subjective sleep quality, sleep efficiency, sleep duration, and emotional symptoms in insomnia populations (22, 61, 62). Acupuncture has also been explored in MCI, with preliminary randomized or pilot evidence suggesting potential benefits for global cognition or memory-related outcomes (23, 24, 63–65). However, direct evidence for acupuncture in patients with both insomnia and cognitive impairment remains limited. To our knowledge, only one prior study has specifically examined acupuncture for this comorbid condition, and that study was limited by the absence of a sham-acupuncture control, small sample size, lack of randomization, and insufficient objective measurements (25). The present protocol was therefore designed to address this evidence gap using random allocation, a sham-acupuncture comparator, participant and assessor blinding, a prespecified primary endpoint, objective sleep assessment, and exploratory neurophysiological measures.

An important feature of the revised protocol is the prespecification of a single primary endpoint: the between-group difference in the change in PSQI total score from baseline to post-treatment. This choice reflects the clinical focus of the intervention on subjective sleep quality, which is central to chronic insomnia and has been widely used in insomnia research (35, 36). The ISI is retained as a key secondary sleep outcome because it captures insomnia severity and related daytime impact over the preceding two weeks (37). The MoCA-B and MMSE are included as key secondary cognitive outcomes rather than co-primary endpoints. This hierarchy is intended to reduce multiplicity risk and to avoid overinterpreting cognitive changes in a trial powered primarily for sleep improvement. The MoCA-B is particularly relevant for Chinese and low-education populations (38), while the MMSE provides a familiar global cognitive screening measure (39, 40).

Objective PSG parameters will complement subjective outcomes by characterizing sleep architecture, sleep efficiency, sleep latency, wake after sleep onset, arousal, and sleep-stage distribution according to standardized scoring rules (41, 42). ADL, executive-function tests, mood scales, fatigue and sleepiness scales, feasibility/acceptability indices, and safety outcomes are included to characterize broader clinical effects, while resting-state EEG is explicitly exploratory and will be used to generate mechanistic hypotheses rather than confirm efficacy.

The acupoint prescription is based on previous clinical studies, systematic evidence, and clinical experience (31–33). The selected acupoints include scalp and head points related to cognitive and arousal regulation, such as Baihui (GV20), Sishencong (EX-HN1), Fengchi (GB20), Shenting (GV24), and Yintang (GV29), as well as points frequently used for sleep regulation, including Anmian (Extra), Shenmen (HT7), Sanyinjiao (SP6), Shenmai (BL62), and Zhaohai (KI6). Prior studies have reported cognitive or sleep-related effects for protocols involving Baihui, Sishencong, Fengchi, Shenting, Xuanzhong, Anmian, Shenmen, Zhaohai, and Shenmai (64–67). Mechanistic studies and related clinical observations suggest that acupuncture may influence sleep and cognitive symptoms through neuroendocrine, inflammatory, brain-gut, and neural-network pathways (21, 63–65, 68). Nevertheless, because the current trial is powered for a clinical sleep outcome and not for mechanistic endpoints, EEG findings will be interpreted cautiously as exploratory.

Sham-controlled acupuncture trials require careful interpretation. The Streitberger-type non-penetrating placebo needle offers a credible method for controlling for attention, treatment setting, ritual, and patient-practitioner interaction (34). However, sham acupuncture is not necessarily physiologically inert, and contextual effects may be substantial in acupuncture research (69, 70). This issue is particularly relevant in a Chinese clinical setting, where prior familiarity with acupuncture and cultural expectations may influence treatment credibility and subjective outcomes. The present trial attempts to mitigate these concerns by using the same acupoint locations, session frequency, treatment duration, room setting, fixation procedures, and standardized communication in both groups, and by formally assessing participant blinding. Even so, any between-group effect should be interpreted as the effect of acupuncture relative to a credible non-penetrating sham procedure, rather than as a pure separation of specific and non-specific therapeutic components.

This protocol has several strengths. First, it focuses on a clinically relevant comorbid population (chronic insomnia with MCI) that has been underrepresented in acupuncture research. Second, it uses a methodologically rigorous design—random allocation, a credible non-penetrating sham comparator, participant and assessor blinding, and a single prespecified primary endpoint (PSQI). Third, the outcome strategy combines validated patient-reported sleep measures with objective polysomnography, cognitive screening, executive-function testing, mood and daytime-functioning scales, feasibility/acceptability indices, and structured safety monitoring. Fourth, measurement-instrument selection was guided by COSMIN principles for patient-reported outcomes (53), while clinician-administered cognitive tests were justified by prior Chinese-population validation. Finally, exploratory resting-state EEG analyses are included with prespecified mechanistic hypotheses, allowing the trial to generate testable mechanistic evidence for future research.

Several limitations should also be acknowledged. First, this is a single-center trial conducted in a Chinese/Asian population, and all participants are required to be right-handed; therefore, generalizability to other ethnic groups, health-care systems, and left-handed participants may be limited. Second, acupuncture familiarity and cultural expectations may affect treatment credibility and subjective outcomes despite the use of a sham procedure and blinding assessment. Third, the sample size is powered for the PSQI primary endpoint and is not intended to provide definitive evidence for cognitive improvement, PSG changes, or EEG mechanisms. Fourth, the 1-month follow-up is relatively short for assessing sustained cognitive effects or conversion-related outcomes in MCI. Fifth, repeated administration of cognitive and executive-function tests may introduce practice effects, and the study does not stratify participants by MCI subtype, Alzheimer’s disease biomarkers, medication burden, or ApoE status. Finally, patient and public involvement was not incorporated into the protocol design. These limitations indicate that future multicenter, multiethnic trials with longer follow-up, biomarker-informed subgroup analyses, and stronger patient involvement will be needed to confirm and extend the findings.

If the present trial demonstrates that acupuncture produces clinically meaningful improvements in sleep quality without significant adverse events in patients with chronic insomnia and MCI, it could offer an accessible non-pharmacological option for clinicians managing this comorbid population, particularly in settings where CBT-I is not readily available. The trial would also provide proof-of-concept evidence and effect-size estimates to inform the design of larger confirmatory multicenter trials.

In conclusion, this protocol describes one of the few methodologically rigorous, sham-controlled randomized clinical trials specifically targeting chronic insomnia with comorbid MCI. By prioritizing a single sleep-related primary endpoint, applying a structured hierarchy of secondary and exploratory outcomes, and combining subjective, objective, and neurophysiological measures, the trial is designed to generate clinically interpretable evidence on acupuncture for this comorbid population and to inform the design of larger confirmatory trials.
